# Optimize speckle-tracking echocardiography screening in CKD patients: a TyG index-based nomogram model

**DOI:** 10.3389/fcvm.2026.1776867

**Published:** 2026-07-03

**Authors:** Chanchengman Wong, Qing Ye, Ting Xie, Junhua Chen, Xingchu Zhang, Jieqiao Chen, Yongquan Huang, Yin Huang

**Affiliations:** 1 Department of Cardiology, The Fifth Affiliated Hospital of Sun Yat-Sen University, Zhuhai, China; 2 Department of Ultrasound, The Fifth Affiliated Hospital of Sun Yat-Sen University, Zhuhai, China; 3 Center of Interventional Medicine, The Fifth Affiliated Hospital of Sun Yat-sen University, Zhuhai, China

**Keywords:** chronic kidney disease, global longitudinal strain, nomogram model, risk stratification, triglyceride-glucose index

## Abstract

**Background:**

Chronic kidney disease (CKD) patients with worsening left ventricular (LV) systolic function face significantly poorer clinical outcomes. While speckle-tracking echocardiography (STE)-derived global longitudinal strain (GLS) can sensitively detect early LV systolic dysfunction, indiscriminate routine STE screening in CKD populations may lead to unnecessary healthcare resource utilization. Therefore, identifying CKD patients at high risk of concurrent abnormal GLS in clinical practice may help optimize the selective use of STE. Notably, the triglyceride-glucose (TyG) index, as a reliable and readily accessible indicator for assessing insulin resistance, has demonstrated a close association with GLS in multiple studies. Therefore, this study aimed to develop a TyG index-based model to predict the risk of abnormal GLS in CKD patients, thereby providing a basis for optimizing screening strategies using STE.

**Methods:**

This prospective cross-sectional study enrolled CKD patients from the Fifth Affiliated Hospital of Sun Yat-sen University between July 2023 to July 2024. All CKD patients underwent clinical examination, biochemistry measurement and transthoracic echocardiography.

**Results:**

Among 260 initially screened CKD patients, 212 were included after exclusions (median age: 47 years, male: 58%). Multivariable analysis identified male sex (OR = 2.77), diastolic blood pressure (OR = 1.07), high-density lipoprotein (OR = 0.36) and TyG index (OR = 4.55) as independent predictors of reduced GLS (< 20%). The developed nomogram model demonstrated robust predictive performance (AUC 0.838-0.842) and clinical utility across multiple risk thresholds in both training and validation cohorts.

**Conclusion:**

The developed nomogram model could serve as an effective screening tool to optimize STE utilization in CKD patients, thereby conserving medical resources.

## Introduction

Chronic Kidney Disease (CKD) represents a significant global public health challenge, with epidemiological studies indicating its prevalence in approximately 10% of the global population (1). In recent years, the cardio-renal crosstalk mechanism has garnered increasing attention (2). Through neurohormonal activation, toxin accumulation, and other pathways, many CKD patients develop structural and functional cardiac abnormalities, particularly progressive deterioration of left ventricular systolic function (3). Notably, left ventricular systolic dysfunction can also exacerbate renal functional decline by impairing renal perfusion and worsening volume overload, thereby establishing a vicious cycle (4). This pathophysiological interplay contributes to cardiovascular disease being the most prevalent complication and the leading cause of mortality in CKD patients (5). Therefore, early detection of left ventricular systolic dysfunction carries significant prognostic value for improving outcomes in this population.

In clinical settings, speckle tracking echocardiography (STE) has been recommended by international guidelines as a key tool for detecting subclinical systolic dysfunction due to its high sensitivity (6). In particular, global longitudinal strain (GLS) derived from STE is a sensitive indicator for detecting left ventricular systolic dysfunction (7), providing critical evidence for cardiovascular risk stratification in patients with CKD (8). However, the application of STE in clinical practice is relatively time-consuming, especially in image acquisition and post-processing. Therefore, the routine, indiscriminate application of STE to CKD patients may result in the inefficient allocation of healthcare resources. There is an urgent need for efficient screening strategies to identify individuals at high risk who would benefit most from further STE evaluation.

The triglyceride-glucose (TyG) index, as a reliable and readily accessible indicator for assessing insulin resistance, has demonstrated a strong association with GLS in multiple studies (9–11). Therefore, the present study aimed to develop a TyG index-based model to predict the risk of abnormal GLS in CKD patients. Such a model could effectively triage these patients, ensuring that STE examination is reserved for those at high-risk.

## Methods

### Study population

From July 2023 to July 2024, patients were prospectively recruited from the nephrology department of the Fifth Affiliated Hospital of Sun Yat-sen University. Eligible participants included those with a diagnosis of CKD, age ≥ 18 years old and LVEF ≥ 50%. The exclusion criteria included individuals with atrial fibrillation, significant valvular abnormality (moderate to severe), cardiomyopathy, pericardial disease, congenital heart disease and poor image quality. CKD staging was performed according to the most recent KDIGO guideline (12). This study has obtained approval from the Ethics Committee of the Fifth Affiliated Hospital of Sun Yat-sen University.

### Echocardiographic measurement

**Figure 1:**
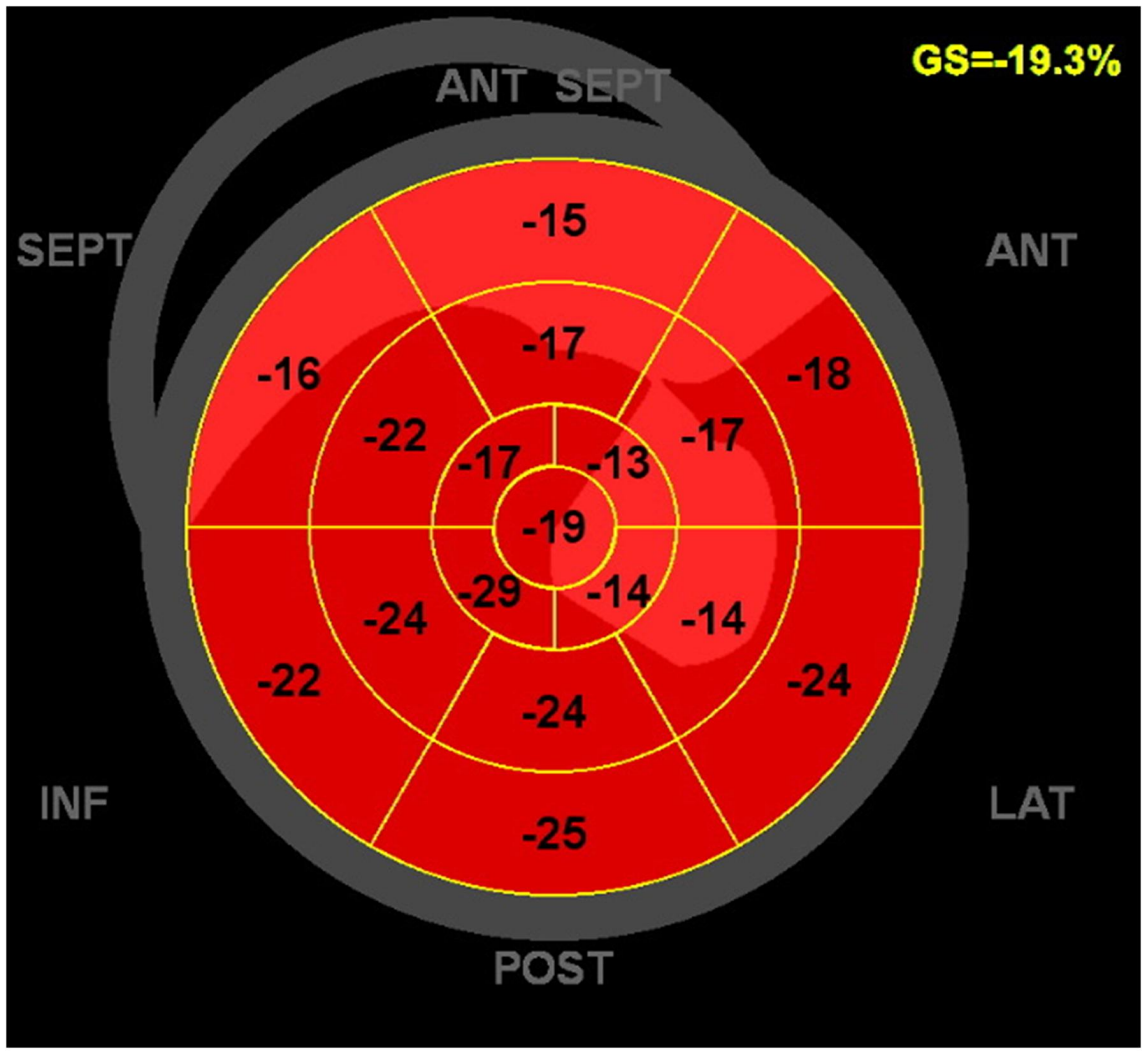
Bull's eyes diagram showing the GLS. GLS, global longitudinal strain.

All echocardiographic views were acquired in accordance with the American Society of Echocardiography guidelines by an experienced sonographer using a GE Vivid E95 system (GE Healthcare, Wauwatosa, WI, USA). A second blinded sonographer performed offline analyses. Left ventricular end-diastolic volume (LVEDV) and end-systolic volume (LVESV) were measured from apical 4- and 2- chamber views using the biplane Simpson's method. Left ventricular ejection fraction (LVEF) was calculated using the formula: LVEF (%) = (LVEDV−LVESV)/LVEDV × 100%. The global longitudinal strain (GLS) was analyzed using speckle-tracking automated function imaging, obtained by averaging the peak systolic strain from all left ventricular segments in apical views (Figure 1), and reported as absolute value (higher absolute value indicates better systolic function). Reduced GLS was defined as an absolute value < 20%, based on the recommendations from the American Society of Echocardiography and the European Association of Cardiovascular Imaging (13, 29).

### Data collection

Demographic characteristics and medication use were obtained from the electronic medical record system of the Fifth Affiliated Hospital of Sun Yat-sen University. Part of laboratory parameters such as fasting plasma glucose (FPG), low-density lipoprotein (LDL), high-density lipoprotein (HDL), serum creatinine (Cr), estimated glomerular filtration rate (eGFR), and fasting triglycerides (FTG) were collected after an overnight fast of at least 8 h. The TyG index was calculated using the formula: TyG = Ln[FTG (mg/dL) × FPG (mg/dL)/2].

### Statistical analysis

Statistical analysis was performed using SPSS version 25.0 (IBM Corp., Armonk, NY, USA), R software version 4.4.1 (R Foundation for Statistical Computing, Vienna, Austria), and GraphPad Prism version 8.0 (GraphPad Software, San Diego, CA, USA). Continuous variables were expressed as mean ± standard deviation for normally distributed data or median (interquartile range, IQR) for non-normally distributed data. Comparisons between groups were made using Student's t-test for normally distributed continuous variables or the Mann–Whitney U test for non-normally distributed data. Categorical variables were presented as frequencies (percentages) and analyzed using the *χ*^2^ test or Fisher's exact test, as appropriate. Univariable logistic regression analysis was first conducted to explore potential associations between candidate variables and reduced GLS (<20%). Variables with a *P* value < 0.05 in the univariable analysis were entered into a multivariate logistic regression model using the forward: conditional method to identify independent predictors associated with impaired GLS. All statistical tests were two-sided, and a *P* value < 0.05 was considered statistically significant.

## Results

### Patient selection

**Figure 2:**
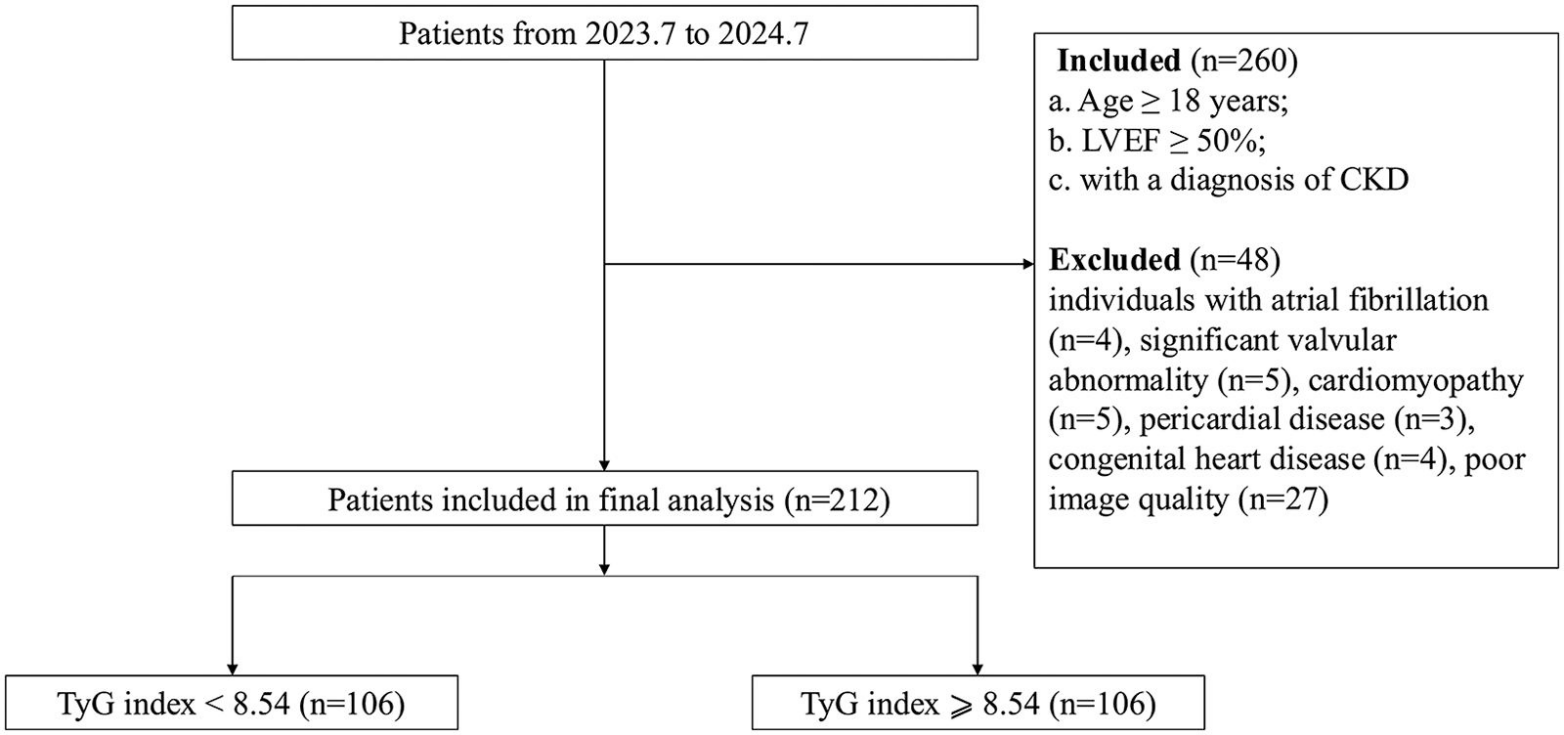
Study flow diagram. CKD, chronic kidney disease; TyG, triglyceride-glucose index.

Among the initial 260 patients meeting inclusion criterias, 48 were excluded from the final analysis due to predefined exclusion criterias, specifically: atrial fibrillation (4 cases), pericardial disease (3 cases), significant valvular abnormalities (5 cases), cardiomyopathy (5 cases), congenital heart disease (4 cases), and poor image quality (27 cases) (Figure 2). A total of 212 CKD patients were included in the analysis. These patients had a median age of 47 years (IQR: 18 years) and were predominantly male (58%).

### Demographic and clinical characteristics

Participants were divided into two groups based on the median TyG index: Group 1 (106 subjects, TyG < 8.54) and Group 2 (106 subjects, TyG ≥ 8.54). Baseline characteristics are detailed in Table 1. Compared to Group 1, Group 2 had a higher body mass index (BMI) [24.12 (IQR: 4.29) kg/m^2^ vs. 22.49 (IQR: 3.82) kg/m^2^, *P* = 0.002] and a lower GLS [16.55% (IQR: 3.07%) vs. 18.05% (IQR: 4.82%), *P* < 0.01]. Additionally, significant differences existed between the two groups in diastolic blood pressure (DBP), FPG, FTG, total cholesterol (TC), HDL, and sodium-glucose cotransporter 2 (SGLT-2) inhibitor usage (all *P* < 0.05). No statistical differences were observed between groups for other indicators such as age and gender.

**Table 1:** Baseline characteristics of the study population according to the median of the TyG index.

Variables	Total(*n* = 212)	Group 1(*n* = 106)	Group 2(*n* = 106)	*P* value
TyG	8.54 (0.79)	8.21 (0.41)	8.99 (0.78)	<0.001
General conditions				
Age, years	47.00 (18.00)	46.00 (17.75)	48.00 (17.25)	0.152
Male, *n* (%)	123 (58.00)	38 (35.85)	80 (75.47)	0.546
BMI, kg/m^2^	23.15 (4.31)	22.49 (3.82)	24.12 (4.29)	0.002
SBP, mmHg	130.86 ± 19.64	128.42 ± 20.73	133.31 ± 18.27	0.069
DBP, mmHg	82.45 ± 12.61	80.66 ± 13.28	84.24 ± 11.70	0.039
Smoking, *n* (%)	33 (15.60)	7 (6.60)	26 (24.53)	0.130
Drinking, *n* (%)	9 (4.20)	1 (0.94)	8 (7.55)	0.161
Medical history, *n* (%)				
Hypertension	119 (56.13)	57 (53.77)	62 (58.49)	0.489
Hyperhomocysteinemia	24 (11.32)	13 (12.26)	11 (10.38)	0.665
Hyperlipidemia	53 (25.00)	22 (20.75)	31 (29.25)	0.153
Echocardiography				
LVEF, %	66.00 (11.00)	65.00 (10.25)	66.14 (10.25)	0.341
GLS, %	16.80 (3.97)	18.05 (4.82)	16.55 (3.07)	<0.001
Laboratory text				
Hb, g/dL	118.57 ± 25.33	115.68 ± 24.17	121.46 ± 26.23	0.097
Urea, mmol/L	10.61 (15.11)	9.96 (16.73)	10.67 (12.80)	0.488
Cr, μmol/L	209.95 (771.18)	153.90 (781.50)	236.55 (771.38)	0.154
FPG, mg/dL	85.86 (20.52)	80.10 (14.99)	92.52 (24.30)	<0.001
TC, mmol/L	4.44 (1.52)	4.20 (4.69)	4.78 (1.84)	0.005
LDL, mmol/L	2.58 (1.18)	2.52 (1.15)	2.59 (1.30)	0.145
HDL, mmol/L	1.11 (0.49)	1.24 (0.40)	1.02 (0.41)	<0.001
FTG, mg/dL	117.84 (91.48)	85.06 (25.03)	174.54 (98.12)	<0.001
eGFR, mL/min/1.73m^2^	30.09 (70.45)	36.74 (79.15)	23.47 (57.01)	0.161
Cardiovascular medications, *n* (%)				
Statins	90 (42.45)	39 (36.79)	51 (48.11)	0.095
Beta-blockers	45 (21.23)	24 (22.64)	21 (19.81)	0.614
ACEI/ARB/ANRI	107 (50.47)	53 (50.00)	54 (50.94)	0.891
SGLT-2i	20 (9.43)	4(3.77)	16(15.09)	0.005
CCB	92(43.40)	41(38.68)	51(48.11)	0.166

TyG, triglyceride-glucose; BMI, body mass index; SBP, systolic blood pressure; DBP, diastolic blood pressure; LVEF, left ventricular ejection fraction; GLS, global longitudinal strain; Hb, hemoglobin; Cr, creatinine; FPG, fasting plasma glucose; TC, total cholesterol; LDL, low-density lipoprotein; HDL, high-density lipoprotein; FTG, fasting triglyceride; eGFR, estimated glomerular filtration rate; ACEI, angiotensin converting enzyme inhibitor; ARB, angiotensin receptor blocker; ARNI, angiotensin receptor-neprilysin inhibitors; SGLT-2i, sodium-glucose co-transporter-2 inhibitors; CCB, calcium channel blocker.

### Logistic regression analysis

Using reduced GLS (< 20%) as the dependent variable, univariable logistic regression analysis (Table 2) revealed significant associations between reduced GLS and male sex, BMI, systolic blood pressure (SBP), DBP, serum creatinine, and TyG index (all *P* < 0.05). Multivariable analysis further identified male sex (OR = 2.77, 95% CI: 1.04–7.36, *P* = 0.041), DBP (OR = 1.07, 95% CI: 1.03–1.12, *P* < 0.001), and TyG index (OR = 4.55, 95% CI: 1.80–11.47, *P* = 0.001) as independent predictors of reduced GLS. A sensitivity analysis was performed by applying a more stringent threshold for impaired GLS (≤ 18%). As shown in Supplementary Table 1, the multivariable logistic regression analysis confirmed that male sex (OR = 3.23, 95% CI: 1.71–6.09, *P* < 0.001), SBP (OR = 1.04, 95% CI: 1.02–1.05, *P* < 0.001), and the TyG index (OR = 2.32, 95% CI: 1.35–4.00, *P* = 0.002) remained significant independent predictors. These findings indicate that the predictive value of the TyG index and male sex is robust across different diagnostic thresholds for subclinical myocardial dysfunction.

**Table 2:** Logistic regression for factors associated with reduced GLS.

Variables	Univariable analysis			Multivariable analysis		
	OR	95% CI	*P* value	OR	95% CI	*P* value
Age	0.98	0.95–1.01	0.168			
Male	4.68	2.05–10.67	<0.001	2.77	1.04–7.36	0.041
BMI	1.25	1.09–1.44	0.002			
SBP	1.05	1.02–1.07	<0.001			
DBP	1.09	1.04–1.13	<0.001	1.07	1.03–1.12	<0.001
Smoking	2.01	0.58–7.03	0.272			
Drinking	0.63	0.13–3.18	0.577			
Hypertension	1.93	0.91–4.08	0.088			
Hyperlipidemia	1.32	0.54–3.25	0.542			
Hyperhomocysteinemia	2.17	0.49–9.71	0.310			
SGLT-2i	3.80	0.49–29.41	0.201			
Hb, g/dL	1.02	1.00–1.03	0.055			
Urea, mmol/L	1.00	0.998–1.003	0.759			
Cr, μmol/L	1.001	1.000–1.002	0.010			
FPG, mg/dL	1.02	1.00–1.05	0.086			
TC, mmol/L	0.95	0.76–1.20	0.681			
LDL, mmol/L	0.92	0.70–1.19	0.514			
HDL, mmol/L	0.15	0.06–0.38	<0.001	0.36	0.11–1.16	0.088
FTG, mg/dL	1.02	1.01–1.02	0.001			
eGFR, mL/min/1.73m^2^	0.99	0.98–1.00	0.002			
TyG	5.42	2.43–12.08	<0.001	4.55	1.80–11.47	0.001

GLS, global longitudinal strain; BMI, body mass index; SBP, systolic blood pressure; DBP, diastolic blood pressure; SGLT-2i, sodium-glucose co-transporter-2 inhibitors; Hb, hemoglobin; Cr, creatinine; FPG, fasting plasma glucose; TC, total cholesterol; LDL, low-density lipoprotein; HDL, high-density lipoprotein; FTG, fasting triglyceride; eGFR, estimated glomerular filtration rate; TyG, triglyceride-glucose.

Sex was coded as 0 for female and 1 for male.

### Baseline characteristics: training vs. validation sets

Patients were randomly divided into training and validation sets at a 7:3 ratio. As detailed in Table 3, baseline characteristics demonstrated good balance between the two cohorts (*P* > 0.05). Notably, although there was difference in HDL level between groups, the validity of the data partitioning is supported by the balanced distribution of other clinically relevant characteristics.

**Table 3:** Distribution of baseline characteristics across training and validation sets.

Variables	Training Set (*n* = 149)	Validation Set (*n* = 63)	*P* value
Age, years	48.00 (19.00)	45.52 ± 11.61	0.560
Male, *n* (%)	86.00 (57.70)	37.00 (58.70)	0.891
BMI, kg/m^2^	22.98 (4.31)	23.81 (4.05)	0.391
SBP, mmHg	129.77 ± 19.87	133.44 ± 19.00	0.471
DBP, mmHg	82.17 ± 12.65	83.10 ± 12.60	0.979
Smoking, *n* (%)	24.00 (16.10)	9.00 (14.30)	0.738
Drinking, *n* (%)	6.00 (4.00)	3.00 (4.80)	0.808
LVEF, %	66.00 (11.00)	66.29 ± 7.02	0.355
GLS, %	17.00 (3.50)	16.57 ± 3.09	0.066
Hb, g/dL	118.99 ± 23.32	117.57 ± 29.73	0.920
Urea, mmol/L	9.27 (13.39)	13.13 (17.28)	0.058
Cr, μmol/L	178.40 (742.80)	382.30 (877.40)	0.110
FPG, mg/dL	85.50 (17.82)	87.84 (27.36)	0.101
TC, mmol/L	4.54 (1.40)	4.24 (1.60)	0.234
LDL, mmol/L	2.62 (1.25)	2.43 (1.03)	0.500
HDL, mmol/L	1.20 (0.48)	1.05 (0.31)	0.005
FTG, mg/dL	121.38 (86.83)	117.84 (100.12)	0.992
eGFR, mL/min/1.73m^2^	33.23 (75.93)	15.13 (60.28)	0.104
TyG	8.52 (0.77)	8.70 ± 0.65	0.313
Statins, *n* (%)	64.00 (69.60)	28.00 (30.40)	0.841
Beta-blockers, *n* (%)	30.00 (65.20)	16.00 (29.70)	0.396
ACEI/ARB/ANRI, *n* (%)	82.00 (75.90)	26.00 (24.10)	0.067
SGLT-2i, *n* (%)	16.00 (80.00)	4.00 (20.00)	0.318
CCB, *n* (%)	59.00(64.80)	32.00(35.20)	0.132

BMI, body mass index; SBP, systolic blood pressure; DBP, diastolic blood pressure; LVEF, left ventricular ejection fraction; GLS, global longitudinal strain; Hb, hemoglobin; Cr, creatinine; FPG, fasting plasma glucose; TC, total cholesterol; LDL, low-density lipoprotein; HDL, high-density lipoprotein; FTG, fasting triglyceride; eGFR, estimated glomerular filtration rate; TyG, triglyceride-glucose; ACEI, angiotensin converting enzyme inhibitor; ARB, angiotensin receptor blocker; ARNI, angiotensin receptor-neprilysin inhibitors; SGLT-2i, sodium-glucose co-transporter-2 inhibitors; CCB, calcium channel blocker.

### Model development and validation

**Figure 3:**
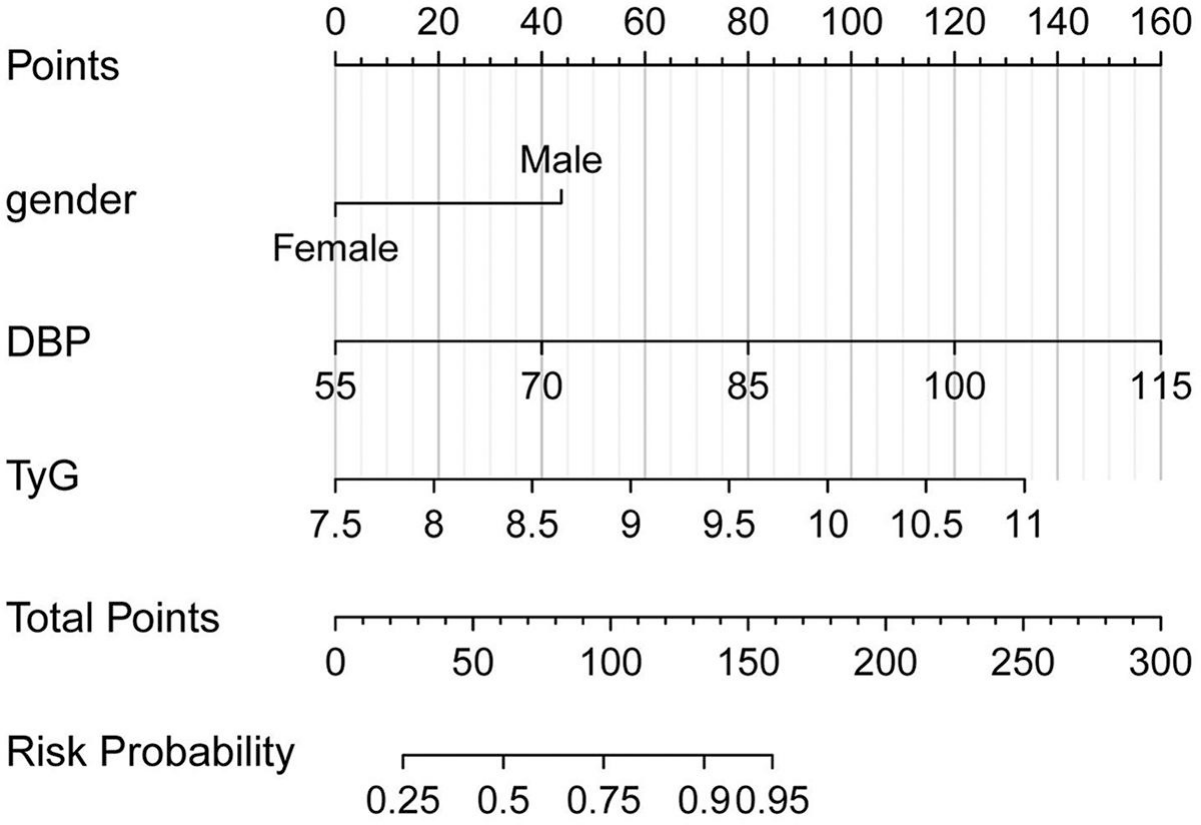
Nomogram model for predicting reduced GLS. DBP, diastolic blood pressure; TyG, triglyceride-glucose; GLS, global longitudinal strain.

**Figure 4:**
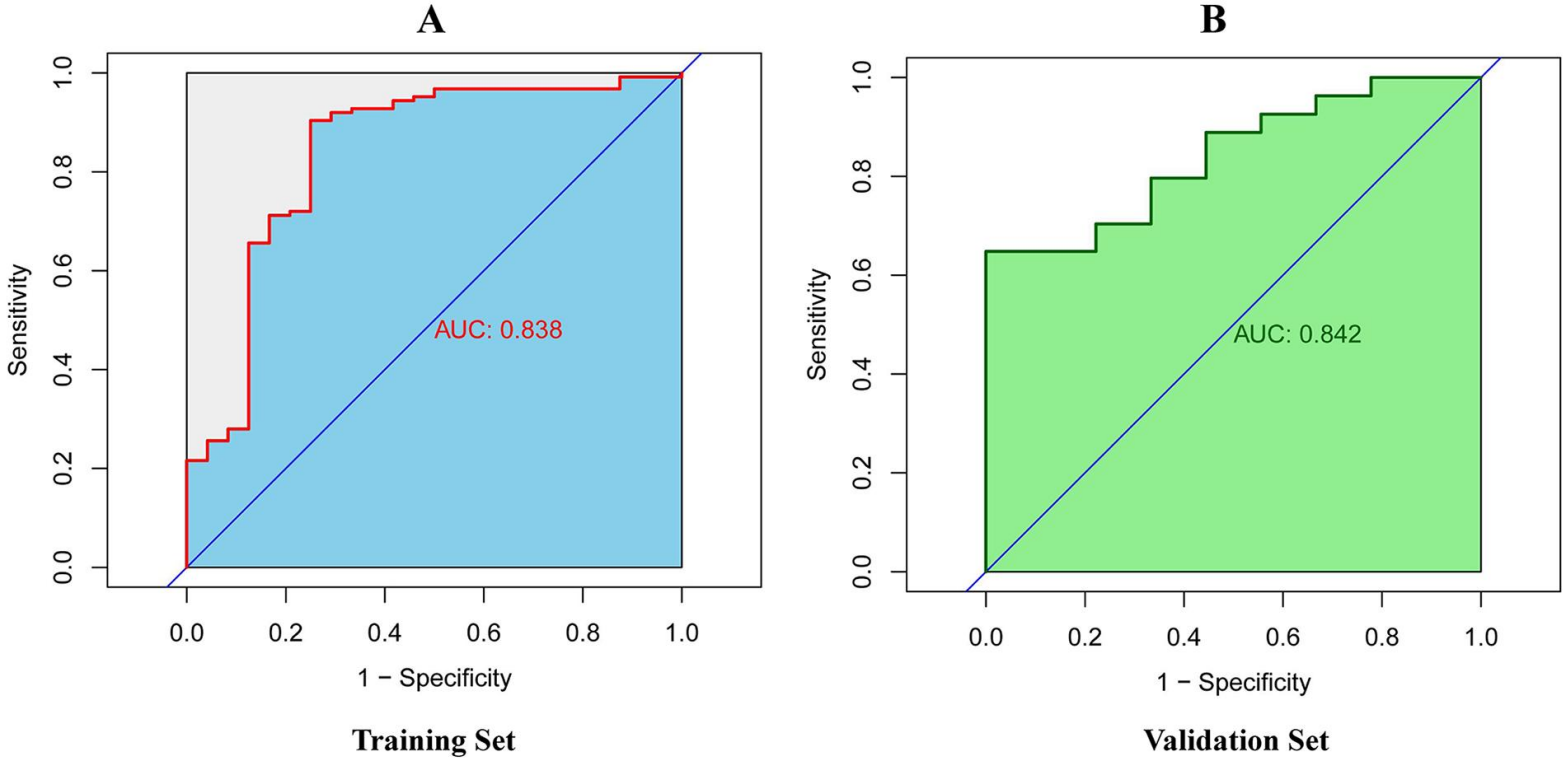
**(A)** Receiver operating characteristic (ROC) curve of the nomogram model in the training set. **(B)** ROC curve of the nomogram in the validation set.

**Figure 5:**
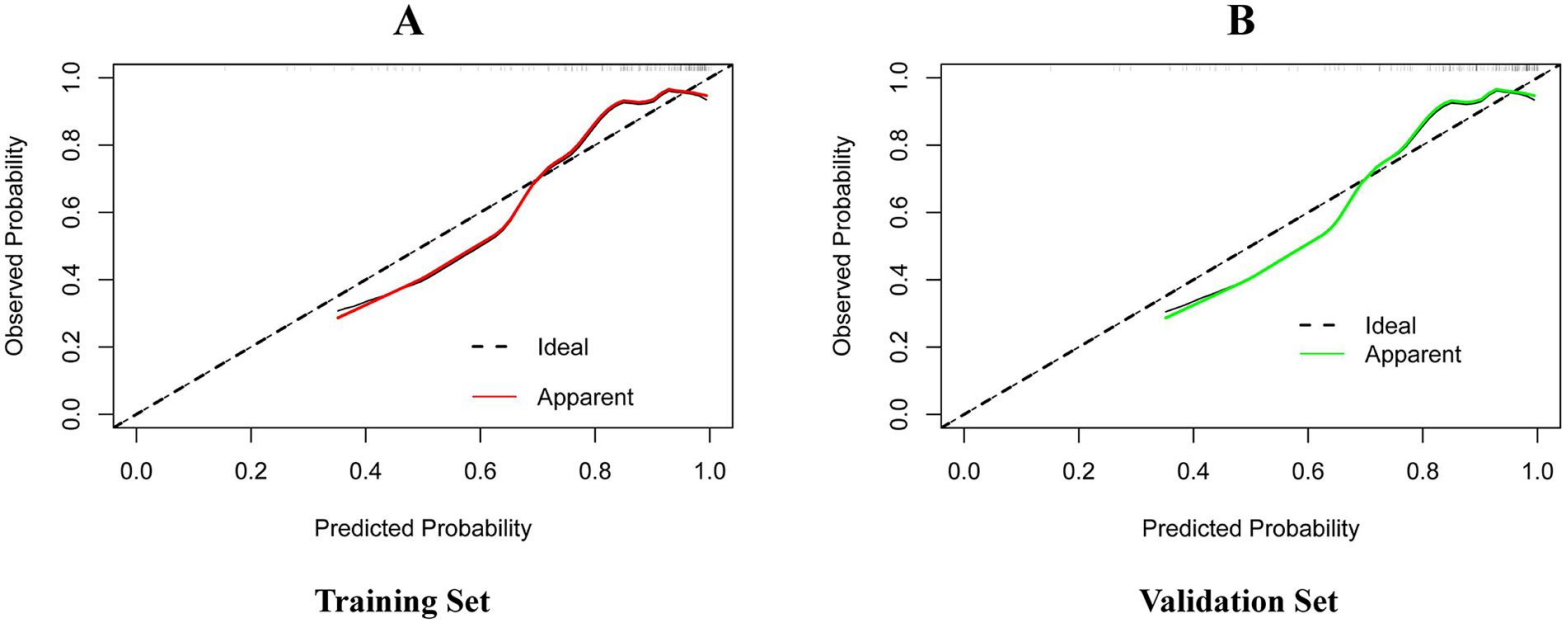
**(A)** Calibration curve of the nomogram model in the training set. **(B)** Calibration curve of the nomogram model in the validation set.

**Figure 6:**
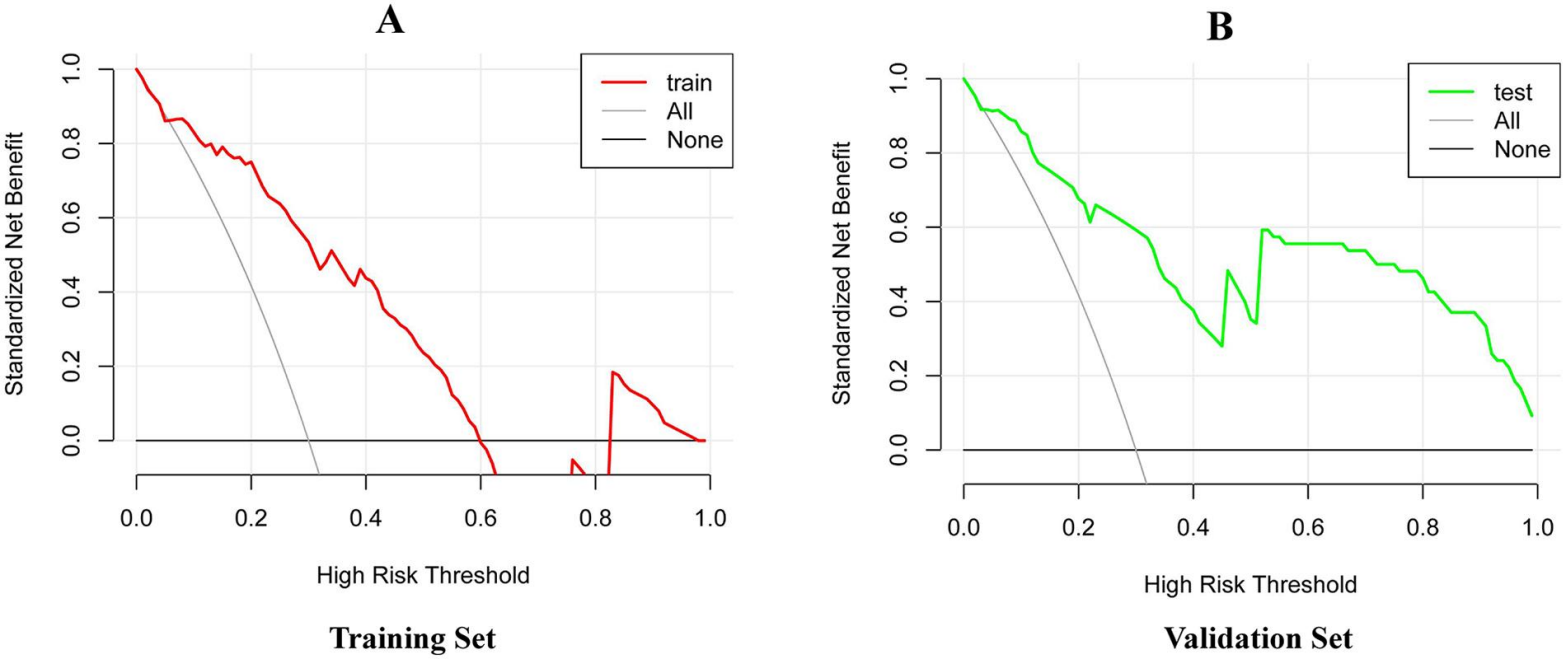
Decision curve analysis of the nomogram model in the training (**A**) and validation (**B**) sets.

The three independent predictors identified through multivariable analysis were used to construct a nomogram model for predicting reduced GLS in the training set (Figure 3). The developed model demonstrated robust predictive performance across both cohorts, with area under the ROC curve (AUC) values of 0.838 (training set) and 0.842 (validation set) (Figure 4), suggesting favorable discriminative ability without evidence of overfitting. As shown in Figure 5, calibration curve analysis revealed that the predicted probabilities exhibited good agreement with actual observations in both sets, validating the model's satisfactory calibration. Decision curve analysis demonstrated that the model yielded significant clinical net benefit across a wide threshold probability range (training set: 0.08–0.60; validation set: 0.08–1.00; Figure 6), confirming its ability to provide reliable support for clinical decision-making. To further evaluate the incremental value of the TyG index, we compared the predictive performance of the basic clinical model with that of the full model including the TyG index. The AUC of the basic clinical model was 0.848 (95% CI: 0.772–0.924). Upon the addition of the TyG index, the AUC improved to 0.866 (95% CI: 0.800–0.932). These results suggest that the TyG index provides an incremental contribution to the discriminative performance of the model.

## Discussion

This study investigated the association between the TyG index and GLS impairment in CKD patients. Our findings demonstrate that the TyG index is not only an independent correlate of abnormal GLS but also, together with gender and DBP, forms a core set of predictive factors. A nomogram model incorporating these variables exhibited favorable predictive accuracy, offering both novel insights and a practical tool for enhancing the assessment of cardiac function in patients with CKD.

The pathophysiological mechanisms underlying increased cardiovascular risk in CKD patients involve complex multisystem interactions. Primarily, progressive renal dysfunction triggers a cascade of compensatory adaptations, including overactivation of the renin-angiotensin-aldosterone system (RAAS), heightened sympathetic nervous system activity, and chronic inflammatory states (14). Concurrently, the accumulation of uremic toxins directly impairs myocardial energy metabolism, while anemia and secondary hyperparathyroidism further exacerbate cardiac workload (15). These pathological changes collectively contribute to vascular endothelial dysfunction, accelerated arterial stiffening, and myocardial remodeling (16, 17).

Insulin resistance (IR), a prevalent pathophysiological state in CKD patients (18), concurrently drives lipid metabolism disorders and chronic inflammation, together forming the pathological basis of cardiac dysfunction in this population (19). At the molecular level, impaired insulin receptor signaling increases adipose tissue lipolysis, releasing excessive free fatty acids into circulation, while reduced lipid uptake capacity in skeletal muscle and liver promotes ectopic lipid deposition in cardiomyocytes (20). Simultaneously, IR activates pro-inflammatory pathways such as IKKβ/NF-*κ*B and JNK, inducing macrophage polarization toward a pro-inflammatory phenotype and the release of cytokines like TNF-α and IL-6, thereby sustaining low-grade systemic inflammation (21). This study confirms the independent association between the TyG index and GLS, providing novel clinical evidence linking metabolic derangements to cardiac impairment in CKD patients.

In the present study, male sex was identified as an independent risk factor associated with impaired GLS (Table 2). Male patients demonstrated a higher risk of GLS impairment, which may be attributed to sex-specific hormonal modulation of myocardial remodeling. Current evidence indicates that estrogen exerts cardioprotective effects, primarily by regulating the expression of cardiomyocyte calcium-handling proteins to improve cardiac function (22). In contrast, androgens may adversely affect cardiac performance by upregulating pro-fibrotic factors in myocardial tissue (23, 24). These findings suggest the necessity of gender-specific intervention strategies in CKD management.

The independent predictive value of DBP underscores the pivotal role of hemodynamic factors in CKD-related cardiac dysfunction. From a hemodynamic perspective, CKD patients typically exhibit markedly increased peripheral vascular resistance in early stages of the disease, primarily due to impaired renal sodium excretion and renin-angiotensin system activation (25). As the lowest arterial pressure during cardiac diastole, DBP serves as a more sensitive indicator of peripheral resistance changes—a key determinant of increased left ventricular afterload and subsequent myocardial remodeling (26). In contrast, SBP, being more influenced by cardiac output and large artery compliance, often remains relatively stable in the early stages of the disease (27). Besides, the unique vascular calcification pattern in CKD further explains DBP's predictive superiority. These patients predominantly develop medial arterial calcification rather than intimal atherosclerosis, a pathological distinction that primarily affects small arteries and microvasculature, leading to pronounced reduction in diastolic vascular compliance (28). Consequently, elevated DBP reflects incipient vascular dysfunction at an earlier stage, making it a more robust predictor of subclinical cardiac impairment compared to SBP changes.

Although previous studies have demonstrated the association between the TyG index and GLS across several disease conditions, this study is the first to develop a nomogram model for predicting abnormal GLS in patients with CKD based on the TyG index. Unlike previous CKD nomogram models that predominantly target renal outcomes or survival, the present study focuses on impaired GLS as an early indicator of subclinical myocardial dysfunction. The model relies solely on routinely available clinical parameters, does not require specialized testing, and demonstrates favorable discrimination and calibration, indicating strong clinical applicability and practicality. By translating the complex predictive relationship into an intuitive scoring system, it substantially enhances assessment efficiency and user accessibility. Our findings confirm that the model effectively identifies high-risk individuals with impaired GLS, thereby providing a quantitative tool to advance the understanding of the TyG index in cardiac function evaluation. With future external multi-center validation and iterative refinement, this model holds promise as a valuable reference for risk stratification in CKD patients.

However, it is important to acknowledge that the optimal GLS cutoff may vary depending on specific populations and clinical contexts. In patients with CKD, subclinical myocardial dysfunction may occur earlier, and a more stringent threshold has been suggested in some studies. To address this potential variability, we performed a sensitivity analysis using GLS ≤ 18%. The results remained consistent, with both male sex and the TyG index confirmed as stable independent predictors at both thresholds, further solidifying our main conclusion. Although DBP was significant in the primary analysis, SBP became significant in the sensitivity analysis. This shift may be attributed to the high collinearity between blood pressure parameters and suggests that systolic afterload may become more prominent as myocardial injury progresses. This evolution does not undermine our findings but rather provides deeper insights into disease progression and confirms that our core model is not dependent on an arbitrarily cutoff (Supplementary Table 1).

This study has several limitations. First, as a single-center cross-sectional study, it is subject to selection bias and does not allow causal inference; the findings should therefore be interpreted as associations only. Second, although CKD staging was performed according to KDIGO guidelines, detailed characterization of the study population (e.g., stage distribution, etiologies, and dialysis status) was not available, which may limit the generalizability of our findings given the clinical heterogeneity of CKD. Third, some potential confounders, including lifestyle factors and medication adherence, were not fully captured, which may have resulted in residual confounding. Finally, the relatively small sample size and lack of external validation may affect the stability and generalizability of the model. Further multicenter and longitudinal studies are warranted.

## Conclusion

The nomogram model based on the TyG index provided a simple and efficient clinical decision support tool for identifying CKD patients with concurrent impaired GLS. This screening approach helps avoid the waste of medical resources. In the future, this tool will be further strengthened through expanding the sample size and multi-center validation.

## Data Availability

The original contributions presented in the study are included in the article/Supplementary Material, further inquiries can be directed to the corresponding authors.
