# Supplementary material for: Optimize speckle-tracking echocardiography screening in CKD patients: a TyG index-based nomogram model
Source: Front Cardiovasc Med. 2026 Jul 3;13:1776867. doi: 10.3389/fcvm.2026.1776867 (PMC13375737; doi:10.3389/fcvm.2026.1776867)
Supplement: Supplementary file 1 [file Supplementaryfile1.docx]

**Supplementary Table 1** Sensitivity analysis of predictors for impaired GLS using a threshold of ≤18%.

| **Variables** | **Univariable analysis** | | |  | | **Multivariable analysis** | | |
| --- | --- | --- | --- | --- | --- | --- | --- | --- |
|  | **OR** | **95% CI** | **P value** |  | **OR** | | **95% CI** | **P value** |
| Age | 1.00 | 0.98-1.03 | 0.764 |  | |  |  |  |
| Male | 3.80 | 2.11-6.83 | <0.001 |  | | 3.23 | 1.71-6.09 | <0.001 |
| BMI | 1.12 | 1.03-1.23 | 0.010 |  | |  |  |  |
| SBP | 1.04 | 1.02-1.06 | <0.001 |  | | 1.04 | 1.02-1.05 | <0.001 |
| DBP | 1.05 | 1.05-1.08 | <0.001 |  | |  |  |  |
| Smoking | 2.05 | 0.88-4.81 | 0.097 |  | |  |  |  |
| Drinking | 1.20 | 0.29-4.93 | 0.803 |  | |  |  |  |
| Hypertension | 1.83 | 1.04-3.21 | 0.036 |  | |  |  |  |
| Hyperlipidemia | 1.13 | 0.59-2.15 | 0.714 |  | |  |  |  |
| Hyperhomocysteinemia | 1.21 | 0.49-2.98 | 0.673 |  | |  |  |  |
| SGLT-2i | 1.88 | 0.66-5.39 | 0.239 |  | |  |  |  |
| Hb, g/dL | 1.003 | 0.99-1.01 | 0.551 |  | |  |  |  |
| Urea, mmol/L | 1.001 | 0.995-1.007 | 0.721 |  | |  |  |  |
| Cr, μmol/L | 1.001 | 1.000-1.002 | 0.004 |  | |  |  |  |
| FPG, mg/dL | 1.008 | 0.997-1.020 | 0.086 |  | |  |  |  |
| TC, mmol/L | 0.91 | 0.76-1.08 | 0.268 |  | |  |  |  |
| LDL, mmol/L | 0.82 | 0.65-1.02 | 0.079 |  | |  |  |  |
| HDL, mmol/L | 0.22 | 0.10-0.47 | <0.001 |  | |  |  |  |
| FTG, mg/dL | 1.005 | 1.001-1.009 | 0.008 |  | |  |  |  |
| eGFR, ml/min/1.73m^2^ | 0.99 | 0.98-0.99 | <0.001 |  | |  |  |  |
| TyG | 2.65 | 1.60-4.40 | <0.001 |  | | 2.32 | 1.35-4.00 | 0.002 |

Abbreviation: GLS: global longitudinal strain; BMI: body mass index; SBP: systolic blood pressure; DBP: diastolic blood pressure; SGLT-2i: sodium-glucose co-transporter-2 inhibitors; Hb: hemoglobin; Cr: creatinine; FPG: fasting plasma glucose; TC: total cholesterol; LDL: low-density lipoprotein; HDL: high-density lipoprotein; FTG: fasting triglyceride; eGFR: estimated glomerular filtration rate; TyG: triglyceride-glucose.

Note: Sex was coded as 0 for female and 1 for male. Impaired GLS was defined as ≤ 18% in this sensitivity analysis.
